# Dynamic change of neutrophil‐to‐lymphocyte ratio and its predictive value of prognosis in acute ischemic stroke

**DOI:** 10.1002/brb3.3616

**Published:** 2024-07-10

**Authors:** Kai Qian, Jie Hu, Chunyan Wang, Chunxiang Xu, Yanguo Chen, Qing Feng, Ya Feng, Yuncheng Wu, Xiaofeng Yu, Qiuhong Ji

**Affiliations:** ^1^ Department of Neurology Affiliated Hospital of Nantong University, Medical School of Nantong University Nantong China; ^2^ Department of Neurology Dongtai People's Hospital Dongtai China; ^3^ Department of Emergency Dongtai People's Hospital Dongtai China; ^4^ Department of Neurology Shanghai General Hospital, Shanghai Jiao Tong University School of Medicine Shanghai China

**Keywords:** acute ischemic stroke, neutrophil‐to‐lymphocyte ratio, prognosis

## Abstract

**Objective:**

The present research aimed to explore the dynamic change of the neutrophil‐to‐lymphocyte ratio (NLR) and its relationship with functional outcome following an acute ischemic stroke (AIS), whether receiving intravenous thrombolysis (IVT) or not.

**Methods:**

We retrospectively analyzed data that were prospectively acquired from patients with AIS treated with IVT or not. For patients receiving IVT, the NLR was based on a blood test performed prior to IVT (d0) and at different time points after disease onset (d1, d3, d7). In addition, in the non‐IVT group, the NLR was obtained at different time points after disease onset (d1, d3, d7). Follow‐ups were performed 3 months after onset via telephone. In addition, a good outcome was defined as a modified Rankin scale (mRS) ≤1; a poor outcome means 2 ≤ mRS ≤ 6.

**Results:**

A total of 204 AIS patients were included in this study. The NLR presented a dynamic change as it increased to its peak at day 1 and gradually declined to its baseline at day 7, no matter whether patients were receiving IVT or not. Patients with poor outcomes have a higher NLR at various time points. The results of multivariate logistic regression analysis demonstrated that the National Institutes of Health Stroke Scale (NIHSS), NLR d1, NLR d3, and NLR d7 were independently associated with functional outcomes. The area under the receiver operating characteristic curve of NLR in predicting outcomes was as follows: NLR d3 demonstrated robust predictive power within the IVT therapy cohort, whereas NLR d7 was predictive in the non‐IVT cohort. However, the most potent predictor emerged as the combination of NIHSS and NLR.

**Conclusion:**

NLR has the potential to predicate diagnosis for AIS, especially when combined with the NIHSS score.

## Graphical abstract

In the AIS patients with IVT or not, we collected blood sample at different timepoints (d0, d1, d3, d7 after disease onset) to calculated the NLR. And we also collected the mRS scores 3 months after disease onset. We aimed to explore the relationship between the NlR and disease outcome, finding a probably biomarker for the diagnosis of AIS.

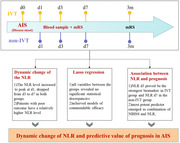

## 1 INTRODUCTION

Acute ischemic stroke (AIS) is the most common type of stroke, accounting for 60%–80% of all strokes. It is one of the most common causes of death or disability, leading to a heavy economic burden globally (He et al., 2024; Mendelson & Prabhakaran, 2021; Tu et al., 2023). Many factors contribute to the prognosis of AIS, including the severity of disease, stenosis or occlusion of intracranial arteries, hypertension, and diabetes (Jolugbo & Ariëns, 2021; Joundi & Menon, 2021; Tu & Wang, 2023; Wang et al., 2024). Recently, cumulating studies have paid more attention to the role of inflammation in AIS, from disease progression to predicative markers of prognosis (Alsbrook et al., 2023; Cai & Hu et al., 2023).

A robust inflammatory response occurs after ischemic stroke (Albaqami et al., 2024; Fu et al., 2023). Ischemic brain tissues release chemokines and cytokines and recruit peripheral circulating neutrophils within hours, followed by lymphocyte (Bui et al., 2022). Yet, these two kinds of leukocytes might exert differential function after AIS and have different influences on the clinical outcomes of patients (Endres & Moro et al., 2022; Sharma et al., 2021; Wang, Cui et al., 2023). Thus, the ratio of neurophil‐to‐lymphocyte (NLR) reflects the peripheral balance between neurophil and lymphocyte levels (Gong et al., 2021). Recent studies have demonstrated that a higher NLR was associated with poststroke depression with a 5.79‐fold increase compared with a lower level (Hu et al., 2020). Moreover, Ki et al. found a higher NLR predicted stroke‐associated pneumonia in patients with AIS (Wang, Wen et al., 2023). In addition, studies have confirmed that NLR is strongly related to the prognosis of infarction (Chen et al., 2023) and thrombo‐inflammatory state and used to be an indicator reflecting the prevalence of intracranial atherosclerosis (Li et al., 2023; Nam et al., 2018). However, these studies only focus on the result of relationship between NRL and AIS and pay no attention to the time it occurs. That is to say, at which time after AIS occurs, the NLR level would be the best choice for a predicative marker.

Thus, we conducted this study to verify the dynamic change of NLR level at different time points after AIS occur and explore the relationship between NLR and the prognosis of AIS, respectively.

## 2 METHODS

### 2.1 Study population

Consecutive AIS patients who underwent treatment with intravenous thrombolysis (IVT) or not at the Dongtai People's Hospital of Jiangsu province from December 2022 to December 2023 were prospectively enrolled in our study. These patients were hospitalized within 24 h from symptom onset. The inclusion criteria for enrollment were (a) older than 18 years, (b) diagnosis of AIS according to the World Health Organization (WHO) criteria and confirmed by magnetic resonance imaging or brain computed tomography and treatment with IVT within 4.5 h of symptom onset, or (c) AIS patients without IVT. Exclusion criteria include (a) evidence of acute infection at admission; (b) cancer, chronic inflammation disease or autoimmune disease, immunosuppressive drug use; disease of blood system; (c) patients who received a bridging therapy consisting of IVT followed by endovascular therapy; and (d) a recent history of major trauma or surgery. We collected 218 patients according to the inclusion and exclusion criteria; a total of 13 patients failed to complete follow‐up, and 1 patient died unexpectedly. Finally, a total of 204 ischemic stroke patients were included in the current study, including 90 patients treated with IVT and 114 AIS patients without IVT. This study was approved by the Ethic Committee of the Dongtai hospital, and all patients or their legally authorized representatives gave informed consent (the ethics approval number was 2022‐dtry‐K‐002).

### 2.2 Clinical measurement

Relevant clinical data were collected from relevant medical records. Baseline information for all patients (age, sex, education level, history of conventional vascular risk factors, such as hypertension, diabetes mellitus, and hypercholesterolemia) and medical history (such as smoking, alcohol consumption, and family history of strokes) were recorded on admission. Stroke severity was assessed by trained neurologists using the National Institutes of Health Stroke Scale (NIHSS) on admission at baseline.

Venous blood samples were collected before IVT (d0) and at different time points after disease onset (d1, d3, d7). The NLR was calculated by dividing the absolute neutrophil count by the absolute lymphocyte count in peripheral blood samples.

### 2.3 Outcome assessments

Functional outcomes were obtained using the modified Rankin scale (mRS) at 3 months after disease onset. Two physicians, trained and certified for data collection, conducted a semiquantitative phone interview for the mRS score. Good functional outcome representing an independent clinical status was defined as an mRS score of 0 or 1, and poor outcome as an mRS score of 2–6.

### 2.4 Statistical analysis

Lasso regression was employed for screening risk factors. The model's predictive performance was evaluated using the C‐index. The C‐index quantifies the agreement between the model's predicted probabilities and the actual outcomes, with values ranging from 0.5 (indicating no predictive capability) to 1.0 (signifying perfect prediction). Multivariable logistic regression analysis was carried out to ascertain whether the NLR independently predicted clinical outcomes, with odds ratios (OR) and 95% confidence intervals (95% CI) being reported. To address potential collinearity among NLR values, a backward stepwise method was utilized in the multivariable logistic regression analysis. Subsequently, the receiver operating characteristic (ROC) curve was used to assess predictive capability and determine the optimal cutoff value for NLR. The Delong method was employed to perform interaction comparisons between the ROC curves, further enhancing the assessment of model performance. The model's performance was validated using the confusion matrix based on the optimal cutoff value. All statistical analyses were performed using the R programming language, with statistical significance defined as *p* values <.05.

## 3 RESULTS

### 3.1 Baseline clinical characteristics of the patients

**FIGURE 1:**
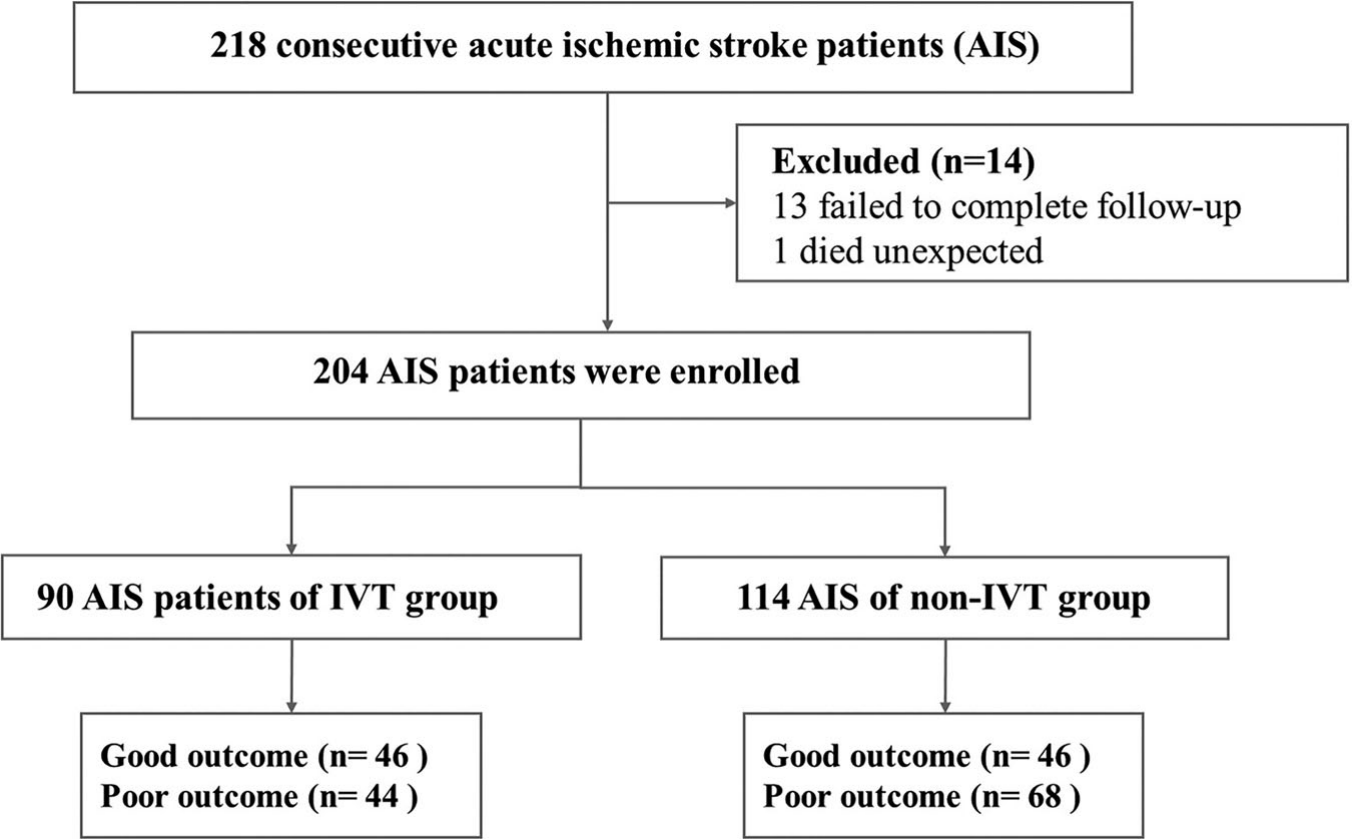
Flow diagram showing the patient selection process. IVT, intravenous thrombolysis.

Two hundred and four ischemic stroke patients (90 vs. 114) with a mean age of 70.99 ± 9.68 vs. 69.72 ± 11.67 were included in the present study (Figure 1). Demographic features and risk factors are shown in Tables 1 and 2. There was no significant difference between the mRS (0–1) and mRS (2–6) in age, gender, smoking, alcohol consumption, hypertension, hyperlipidemia, history of myocardial infarction, history of cerebral infarction, and homocysteine in both IVT and non‐IVT groups. As for patients of IVT, we could observe a significant difference between the groups mentioned above in NLR d1, d3, d7 and the NIHSS score. In addition, the NLR was significantly higher in the poor outcome group than the good outcome group at each time point.

**TABLE 1:** Baseline clinical characteristics of the patients in intravenous thrombolysis (IVT) group.

	Total (*n* = 90)	Good outcome mRS (*n* = 46)	Poor outcome mRS (2–6) (*n* = 44)	p‐Value
**Characteristics mean ± SD/*N* (%)**				
Age, years	70.99 ± 9.68	71.43 ± 9.29	70.52 ± 10.17	.66
Sex	56 (62.22%)	31 (67.39%)	25 (56.82%)	.41
**Vascular risk factors**, ***N* (%)**				
Hypertension	68 (75.56%)	33 (71.74%)	35 (79.55%)	.54
Diabetes mellitus	26 (28.89%)	18 (39.13%)	8 (18.18%)	.05
Active smokers	39 (43.33%)	21 (45.65%)	18 (40.91%)	.81
Drink	31 (34.44%)	18 (39.13%)	13 (29.55%)	.46
History of CI	8 (8.89%)	2 (4.35%)	6 (13.64%)	.15
History of CH	1 (1.11%)	0 (0.00%)	1 (2.27%)	.49
Atrial fibrillation	39 (43.33%)	20 (43.48%)	19 (43.18%)	1.00
Myocardial infarct	4 (4.44%)	2 (4.35%)	2 (4.55%)	1.00
HCY	11.63 (10.02, 13.66)	11.63 (9.84, 13.28)	11.63 (10.09, 13.85)	.45
Systolic pressure	168 (155, 184.5)	168 (157.25, 181.75)	171 (151.75, 191)	.57
Diastolic pressure	92 (83, 102.75)	91.5 (86, 100.75)	93 (76.75, 106.25)	.92
NIHSS	7.5 (5, 12.75)	5.5 (4, 7)	11 (8, 14.25)	.00
DNT	30 (25.25, 49.75)	29 (25.25, 47.75)	38 (25.75, 54.25)	.27
WBC d0	7.53 (5.96, 9.48)	6.83 (5.51, 9.27)	7.86 (6.62, 9.63)	.10
WBC d1	7.47 (6.14, 9.07)	6.67 (5.31, 8.35)	8.29 (7.29, 9.7)	.00
WBC d3	7.22 (5.72, 8.31)	6.38 (5.39, 7.83)	7.76 (6.94, 8.95)	.00
WBC d7	6.63 ± 1.97	6.01 ± 2.06	7.28 ± 1.67	.00
Neutrophil counts (10^9^/L) d0	4.73 (3.75, 6.53)	4.38 (3.62, 6.13)	4.95 (3.96, 6.94)	.08
Neutrophil counts (10^9^/L) d1	5.59 (4.11, 7.02)	4.42 (3.54, 5.88)	6.32 (5.48, 7.98)	.00
Neutrophil counts (10^9^/L) d3	5.08 (3.82, 6.22)	4.3 (3.25, 5.53)	5.65 (4.94, 6.84)	.00
Neutrophil counts (10^9^/L) d7	4.4 (3.13, 5.26)	3.47 (2.72, 4.8)	4.96 (4.21, 5.82)	.00
Lymphocyte counts (10^9^/L) d0	1.61 (1.21, 2.45)	1.68 (1.24, 2.68)	1.41 (1.2, 2.26)	.63
Lymphocyte counts (10^9^/L) d1	1.3 (1.02, 1.68)	1.48 (1.11, 1.83)	1.26 (0.99, 1.52)	.11
Lymphocyte counts (10^9^/L) d3	1.44 (1.1, 1.8)	1.58 (1.24, 1.94)	1.32 (1.05, 1.71)	.03
Lymphocyte counts (10^9^/L) d7	1.44 (1.15, 1.83)	1.49 (1.19, 1.84)	1.39 (1.14, 1.8)	.50
NLR d0	2.93 (1.66, 4.47)	2.5 (1.61, 3.48)	3.58 (1.79, 5.06)	.08
NLR d1	4.25 (2.87, 6.14)	3.39 (2.35, 4.33)	5.66 (4.12, 6.97)	.00
NLR d3	3.37 (2.52, 4.5)	2.64 (2.04, 3.52)	4.22 (3.14, 5.55)	.00
NLR d7	2.82 (2.1, 3.85)	2.34 (1.92, 3.09)	3.8 (2.7, 4.34)	.00
Monocyte counts (10^9^/L) d0	0.42 (0.34, 0.55)	0.42 (0.34, 0.57)	0.42 (0.34, 0.54)	.92
Monocyte counts (10^9^/L) d1	0.46 (0.37, 0.56)	0.42 (0.31, 0.55)	0.47 (0.38, 0.55)	.18
Monocyte counts (10^9^/L) d3	0.5 (0.38, 0.6)	0.44 (0.33, 0.59)	0.54 (0.44, 0.63)	.01
Monocyte counts (10^9^/L) d7	0.43 (0.35, 0.54)	0.4 (0.3, 0.5)	0.46 (0.41, 0.56)	.02
PLT d0	178 (142.25, 208)	185 (137.5, 209.5)	178 (143.75, 205.25)	.73
PLT d1	169 (133.5, 204.5)	171 (132.25, 204)	168.5 (136.5, 205.75)	.68
PLT d3	165 (136.75, 204.25)	162 (132.5, 205.75)	166.5 (139, 191.25)	.90
PTL d7	182.5 (155.25, 208.25)	175.5 (140, 201.5)	190 (165, 211.5)	.22

Abbreviations: CH, cerebral hemorrhage; CI, cerebral infarction; DNT, door‐to‐needle time; HCY, homocysteine; mRS, modified Rankin scale; NIHSS, National Institutes of Health Stroke Scale; NLR, neutrophil‐to‐lymphocyte ratio; PLT, platelet; SD, standard deviation; WBC, white blood cell.

**TABLE 2:** Baseline clinical characteristics of the patients in non‐intravenous thrombolysis (IVT) group.

	Total (*n* = 114)	Good outcome mRS (*n* = 46)	Poor outcome mRS (2–6) (*n* = 68)	*p*‐Value
**Characteristics mean** ± **SD/*N* (%)**				
Age, years	69.72 ± 11.67	68.3 ± 12.21	70.68 ± 11.29	.30
Sex	61 (53.51%)	29 (63.04%)	32 (47.06%)	.14
**Vascular risk factors**, ***N* (%)**				
Hypertension	87 (76.32%)	32 (69.57%)	55 (80.88%)	.24
Diabetes mellitus	32 (28.07%)	5 (10.87%)	27 (39.71%)	.00
Active smokers	37 (32.46%)	19 (41.30%)	18 (26.47%)	.15
Drink	30 (26.32%)	17 (36.96%)	13 (19.12%)	.06
History of CI	13 (11.40%)	4 (8.70%)	9 (13.24%)	.56
History of CH	2 (1.75%)	1 (2.17%)	1 (1.47%)	1.00
Atrial fibrillation	32 (28.07%)	14 (30.43%)	18 (26.47%)	.80
Myocardial infarct	0 (0.00%)	0 (0.00%)	0 (0.00%)	.04
NIHSS	5 (3, 7)	3 (2, 5)	6 (4, 9)	.00
WBCd1	6.88 (5.26, 8.11)	6.42 (5.1, 7.88)	7.01 (5.69, 8.3)	.18
WBCd3	6.64 (5.46, 8.12)	5.97 (4.97, 7.36)	7.04 (5.69, 8.76)	.02
WBCd7	6.38 ± 1.87	5.96 ± 1.71	6.67 ± 1.92	.04
Neutrophil counts (109/L) d1	4.87 (3.44, 6.09)	4.14 (3.37, 5.55)	5.18 (3.72, 6.56)	.02
Neutrophil counts (109/L) d3	4.28 (3.53, 5.96)	3.86 (3.17, 4.69)	4.89 (3.79, 6.55)	.00
Neutrophil counts (109/L) d7	3.99 (2.92, 5.09)	3.47 (2.54, 4.29)	4.28 (3.36, 5.38)	.00
Lymphocyte counts (10^9^/L) d1	1.45 (1.05, 1.79)	1.65 (1.25, 1.92)	1.34 (0.98, 1.67)	.02
Lymphocyte counts (10^9^/L) d3	1.47 (1.17, 1.88)	1.54 (1.21, 1.91)	1.45 (1.15, 1.84)	.54
Lymphocyte counts (10^9^/L) d7	1.54 (1.24, 1.81)	1.61 (1.35, 2)	1.46 (1.22, 1.75)	.06
NLR d1	3.43 (2.15, 5.11)	2.5 (2, 3.97)	4.09 (2.64, 5.9)	.00
NLR d3	2.91 (2.22, 4.09)	2.46 (1.92, 3.27)	3.21 (2.49, 4.72)	.00
NLR d7	2.68 (1.91, 3.77)	2.05 (1.5, 3.07)	3 (2.28, 4.07)	.00

Abbreviations: CI, confidence intervals; mRS, modified Rankin scale; NIHSS, National Institutes of Health Stroke Scale; NLR, neutrophil‐to‐lymphocyte ratio.

### 3.2 Dynamic change of the NLR

**FIGURE 2:**
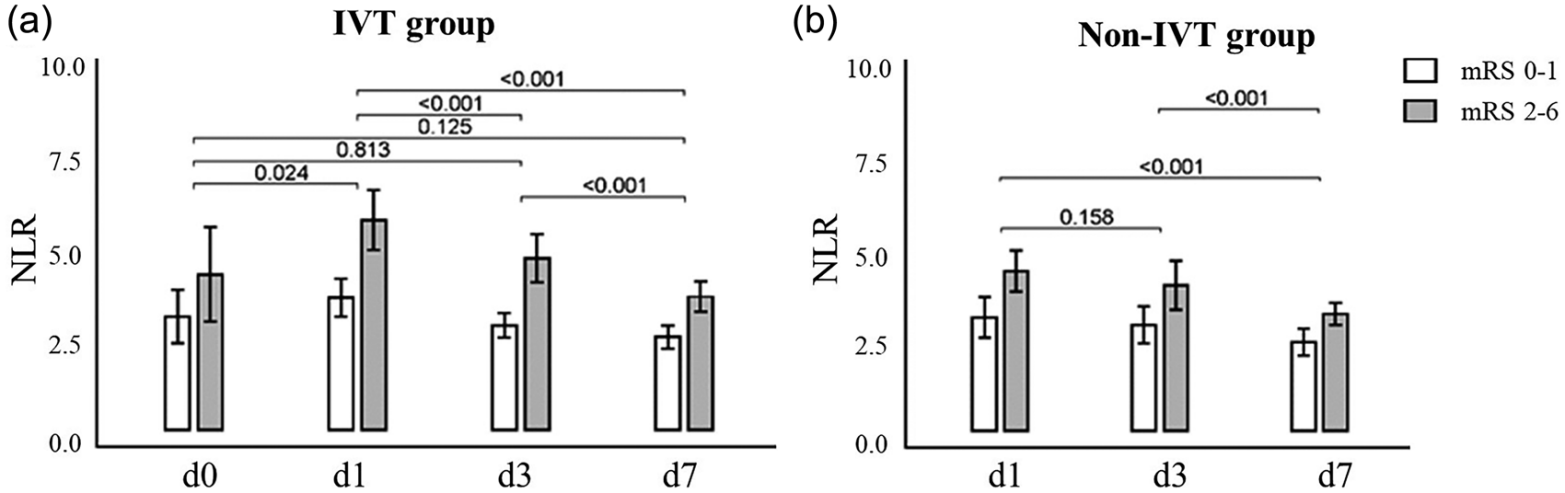
Boxplots to show a dynamic change of neutrophil‐to‐lymphocyte ratio (NLR) within intravenous thrombolysis (IVT) (a) and non‐IVT groups (b).

In this study, we collected blood samples at different time points and calculated the NLR, respectively. From Figure 2, we could see the dynamic change trends of NLR at different time points. Both in IVT and non‐IVT groups, the NLR level increased to its peak at day 1 and dropped gradually to the baseline level from days 3 to 7. In addition, what's evident is that patients with poor outcomes have a relatively higher NLR level (mRS 0–1: 3.158, 3.695, 2.915, 2.589; mRS 2–6: 4.331, 5.843, 4.788, 3.718). Notably, the disparity in NLR dynamics between these mRS categories is less evident in the non‐IVT cohort. Nonetheless, the trend of an augmented NLR in patients with more severe disability (mRS 2–6) is consistently observed (mRS 0–1: 3.159, 2.957, 2.475; mRS 2–6: 4.450, 4.059, 3.261).

### 3.3 Lasso regression

**FIGURE 3:**
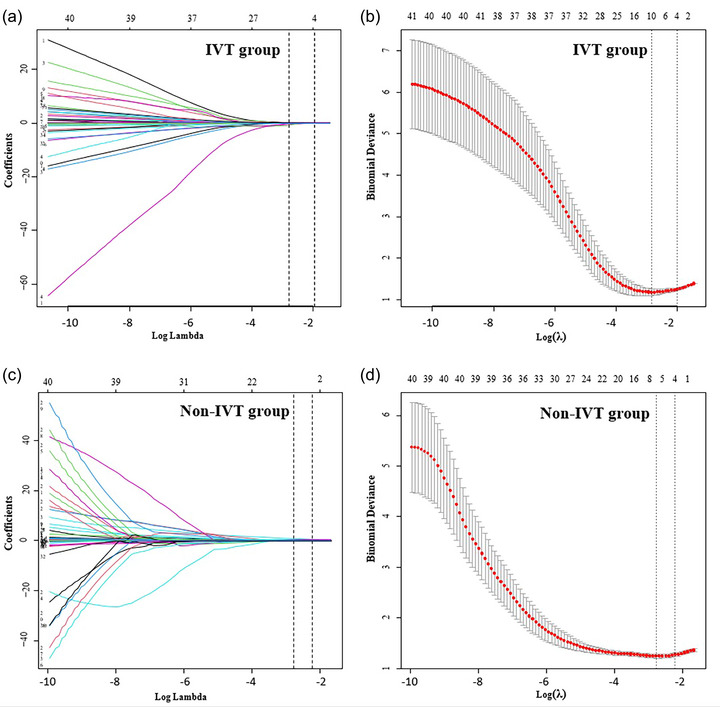
Variable selection based on Lasso regression. (a and c) The changing characteristics of variable coefficients; (b and d) the process of selecting the optimal parameter *λ* in the Lasso regression model by cross‐validation method.

The patient cohort was randomly allocated into two subsets, a training set and a validation set, in an equal distribution ratio of 1:1. Comparative analysis of all variables between the groups revealed no significant statistical discrepancies (*p* > .05), affirming that the stratification of the data was both random and methodologically sound. Lasso regression was employed to refine the selection of parameters, with Figure 3a,c illustrating the dynamic progression of the variable coefficients. The optimization of the model was facilitated by a 10‐fold cross‐validation process, which iteratively refined the analysis. At *λ* values of 0.163 (log *λ* = −0.785) for the thrombolysis group and 0.154 (log *λ* = −0.812) for the non‐thrombolysis group, we achieved models of commendable efficacy characterized by the minimal yet most significant variables (as shown in Figure 2b,d). The C‐index is 0.802 and 0.808 for the validation set in the thrombolysis group and the non‐thrombolysis group, respectively.

### 3.4 Association among NIHSS, NLR, and prognosis

**FIGURE 4:**
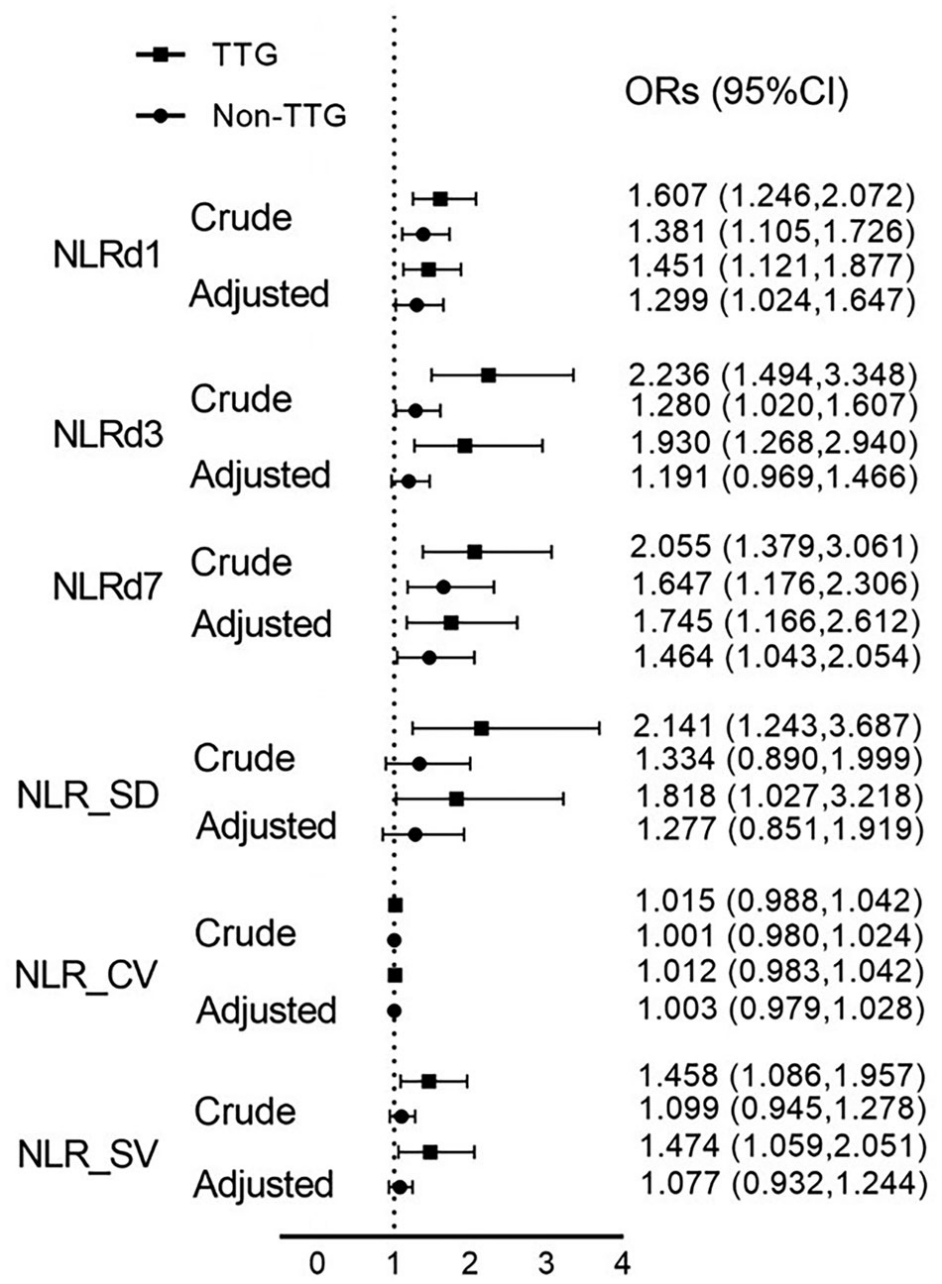
Univariate and multivariate logistic analyses of neutrophil‐to‐lymphocyte ratio (NLR) associated with the modified Rankin scale (mRS) in intravenous thrombolysis (IVT) and non‐IVT groups. (Crude: Univariate logistics regression analyses; adjusted: adjusted for admission National Institutes of Health Stroke Scale [NIHSS] score). 95% CI, 95% confidence intervals; OR, odds ratio.

As shown in Figure 4, univariate and multivariate logistic analyses were conducted by the stepwise method, and NLR d3 (OR: 2.236; 95% CI: 1.494–3.348) proved to be the strongest biomarker in the IVT group and NLR d7 (OR: 1.647; 95% CI: 1.176–2.306) proved to be the strongest in the non‐IVT group. After adjusted for NIHSS, both remained strongest independent predictors in the IVT group (NLR d3: OR: 1.930; 95% CI: 1.268–2.940) and the non‐IVT group (NLR d7: OR: 1.464; 95% CI: 1.043–2.054). In addition, we also observed that NLR_SD, NLR_CV, and NLR_CV lost statistical significance after adjusting for NIHSS (*p* > .05).

**FIGURE 5:**
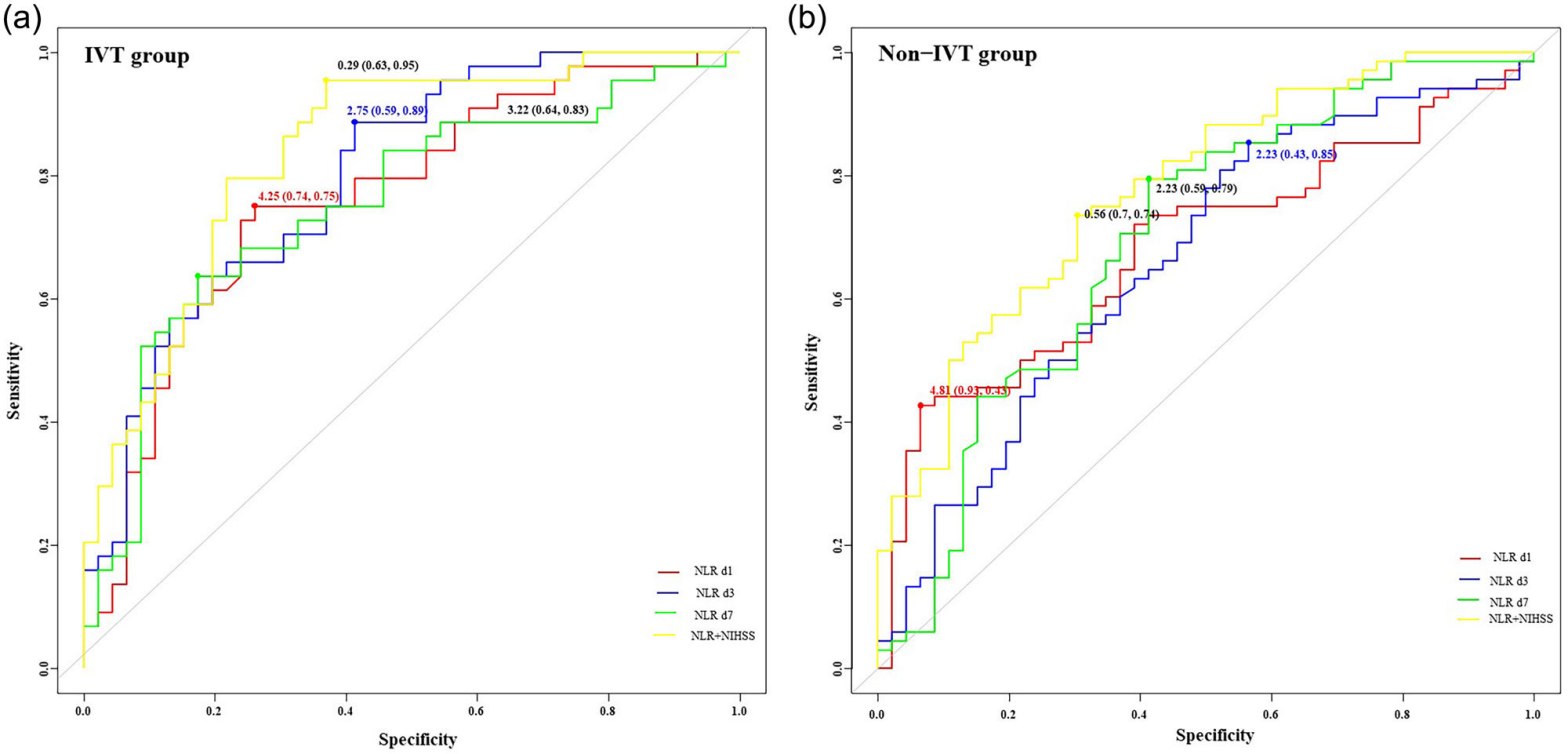
Receiver operating characteristic (ROC) analysis of neutrophil‐to‐lymphocyte ratio (NLR) associated with modified Rankin scale (mRS) in intravenous thrombolysis (IVT) (a) and non‐IVT groups (b).

**FIGURE 6:**
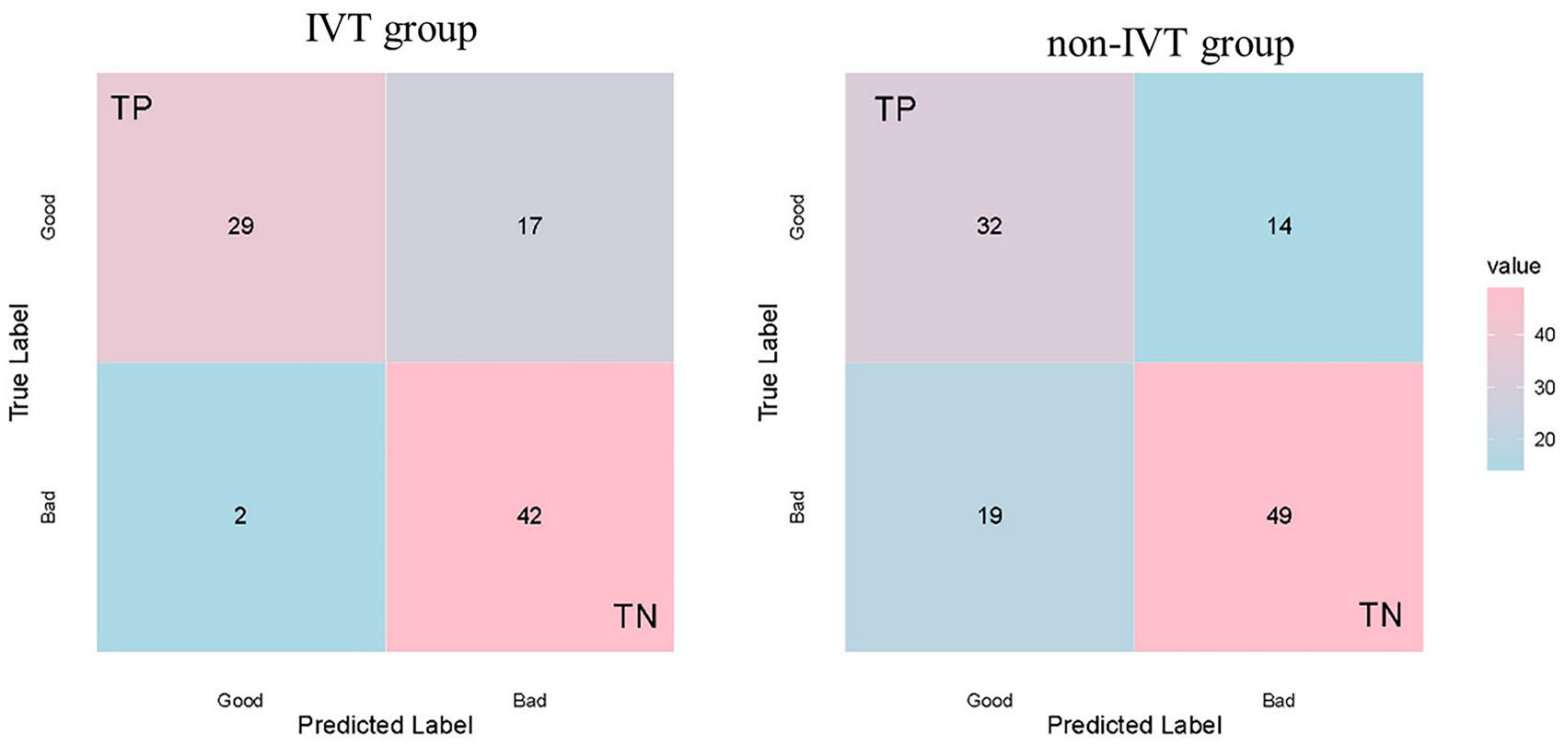
Confusion matrix of the receiver operating characteristic (ROC) model's predicted results in the data.

The ROC curve was utilized to assess the predictive capability of the NLR, along with its cutoff value (Figure 5 and Table 3). Given that the AUCs for NLR_SD, NLR_CV, and NLR_SV were comparatively low (<0.7) and lost statistical significance upon readjustment, further analysis of these metrics was deemed unnecessary and subsequently discontinued. Notably, NLR d3 demonstrated robust predictive power within the IVT therapy cohort, whereas NLR d7 was predictive in the non‐IVT cohort. However, the most potent predictor emerged as the combination of NIHSS and NLR, with projected AUCs of .840 (95% CI: .748–.909) and .777 (95% CI: .690–.850) for the IVT and non‐IVT groups, respectively. The *p*‐values for interaction within the figures represent the statistical significance of the interaction effect between thrombolytic therapy and the respective variables (NIHSS, NLR1d, NLR3d, NLR7d, NLR_SD, NLR_CV, NLR_SV) or the combination of NIHSS and NLR in predicting outcomes. These *p*‐values indicate whether the impact of the variables on outcomes differs significantly between the thrombolytic and non‐thrombolytic therapy groups. The optimal cutoff values were determined to be.29 (specificity.63, sensitivity.95) and.56 (specificity.7, sensitivity.74), respectively. Ultimately, we constructed confusion matrices (Figure 6) using the NIHSS + NLR model, with accuracies and precisions of.789,.630 and.711,.696 in the IVT and non‐IVT groups, respectively.

**TABLE 3:** Receiver operating characteristic (ROC) of neutrophil‐to‐lymphocyte ratio (NLR) associated with modified Rankin scale (mRS) in thrombolytic and non‐thrombolytic therapy groups.

	IVT group			non‐IVT group		
**Model**	AUC (95% CI)	*p*‐Value	*p‐*Value for interaction	AUC (95% CI)	*p*‐Value	*p‐*Value for interaction
NIHSS	.795 (.697–.873)	<.0001	.2390	.746 (.656–.823)	<.0001	.3237
NLR d1	.764 (.663–.847)	<.0001	.0496	.682 (.588–.766)	.0003	.0374
NLR d3	.800 (.702–.877)	<.0001	.1976	.662 (.567–.748)	.0021	.0175
NLR d7	.755 (.653–.840)	<.0001	.0565	.697 (.604–.780)	.0002	.1026
NLR_SD	.697 (.591–.790)	.001	.0051	.588 (.492–.679)	.1023	.0009
NLR_CV	.561 (.452–.665)	.330	.0001	.501 (.406–.596)	.9906	< .0001
NLR_SV	.656 (.548–.753)	.009	.0044	.588 (.492–.679)	.1052	.0013
NIHSS + NLR	.840 (.748–.909)	<.0001	/	.777 (.690–.850)	<.0001	/

Abbreviations: 95% CI, 95% confidence intervals; IVT, intravenous thrombolysis; NIHSS, National Institutes of Health Stroke Scale.

## 4 DISSCUSION

Although previous studies have analyzed the association of the NLR with the severity of ischemic stroke (Poyraz & Vupa Çilengiroğlu, 2023) and the significance of the NLR in predicting hemorrhagic transformation after thrombolysis (Xie et al., 2023), the current study included the dynamic change of NLR and its predictive value for the diagnosis of AIS. It was found that AIS patients who underwent IVT treatment presented an increase of NLR levels after disease onset. In addition, this increase reached to its peak at day 1, then decreased gradually at day 3, and returned to the baseline level at day 7. In the non‐thrombolytic treatment group, we could see a similar trend of NLR. Moreover, we demonstrated that a higher level of NLR is closely related to poor prognosis. In addition, the NLR of day 3 has the highest predictive value. This predictive value increased when combined with the NLR and NIHSS scores. It is believed that the NLR at day 3 combined with NIHSS may be readily available prognostic markers for AIS.

Several previous studies have demonstrated the association of NLR with unfavorable functional outcomes in AIS (Chen et al., 2020), acute intracerebral ICH (Zhang et al., 2023), and subarachnoid hemorrhage (Oliveira & Rabelo, 2023). It has been reported that high neutrophil counts have been found at various stages of atherosclerosis, which could damage endothelial cells (Feng et al., 2023). In addition, inflammation represented by NLR was also closely related with a number of vascular risk factors, including diabetes (Dung et al., 2024), hypertension (Xu et al., 2023), and hyperlipidemia (Hashemi Moghanjoughi et al., 2022). Upon AIS occurring, neutrophils appear to be the first leukocytes to reach the ischemic area within 24 h, thus in turn activating the local inflammation process, accompanied with the fast release of pro‐inflammatory cytokines and biomarkers of inflammation (Alsbrook et al., 2023; Feng & Guo et al., 2019). The high level of these inflammatory cytokines further amplifies immune cascade, leading to the production of reactive oxygen species and cellular dysfunction (Matsumoto et al., 2018). Meanwhile, lymphocytes are immune cells for protection. Decrease count of lymphocytes reflects the pathological stress state (Li et al., 2020; Wang et al., 2017).

In our present study of patients with AIS treated with IVT, we found that the NLR level present a dynamic change from day 0 to 7, reaching its peak at day 1. This was consistent with the time of neutrophils migrating to the ischemic brain area (Otxoa‐de‐Amezaga et al., 2019). This increase was alleviated by lymphocyte. In addition, the NLR level returned to baseline at day 7, indicating the end of hyper‐acute phase. The body initiates an immune repair mechanism (Otxoa‐de‐Amezaga et al., 2019), protecting ischemic brain tissue. Consistent with previous studies, our present article also found that patients with poor prognosis had a significantly higher NLR level from days 0 to 7 compared to patients with good prognosis. In addition, this relationship was the most significant at day 3, the AUC of which was approximately 0.8. Moreover, in the non‐thrombolytic group, we could see a similar trend of NLR, though all these patients had not experienced thrombolysis. But all NLR levels are higher in the thrombolysis therapy group than non‐thrombolysis therapy group, indicating that thrombolysis might exert a negative effect on inflammation. In addition, this might partly explain the fact that patients who undergo thrombolysis have an increased risk of complications such as hemorrhage and disease aggravation.

The close relationship between NLR and ischemic stroke severity (defined as NIHSS scores) has been clarified previously (Roman‐Filip & Catană Vlădoiu et al., 2023). In addition, in our present study, we also found that the NLR level combined with NIHSS significantly increased the predicative value for the prognosis of AIS, whether patients underwent thrombolysis or not, especially the level of NLR at day 3 after disease onset.

Taken together, AIS is a life‐threatening disease characterized by high mortality and morbidity. Early determination of the disease prognosis is critical to the management of the disease. Neuro‐inflammation represents a major factor secondary to ischemic stroke onset. NLR is an easily accessible biomarker that has the potential to predicate the prognosis of AIS despite patients experiencing thrombolysis. During the early stage of the disease, NLR level exhibits dynamic change, and the level at day 3 after disease onset has the greatest significance. This may imply a suitable time point to get peripheral blood.

Our present study also has some limitations. First, it lacks a multicenter design. In addition, laboratory data were not available every day within hospital; thus, we could not fully understand the change of NLR in detail. What's more, these findings do not provide any mechanistic evidence, such as sampling analyses or some pro‐inflammatory factor levels. In addition, this is also what we want to do in the near future.

## 5 CONCLUSION

NLR has the potential to predicate diagnosis for AIS, especially when combined with the NIHSS score. Further studies are required to investigate the exact mechanism of this phenomenon, which might provide evidence for future drug development.

## AUTHOR CONTRIBUTIONS

**Kai Qian**: Writing—review and editing; data curation; formal analysis; writing—original draft; software. **Jie Hu**: Data curation; writing—review and editing. **Chunyan Wang**: Data curation; writing—review and editing; writing—original draft. **Chunxiang Xu**: Data curation. **Yanguo Chen**: Data curation. **Qing Feng**: Data curation. **Ya Feng**: Data curation; supervision; writing—review and editing; funding acquisition. **Yuncheng Wu**: Writing—review and editing; supervision. **Xiaofeng Yu**: Writing—review and editing; investigation; methodology; project administration. **Qiuhong Ji**: Writing—review and editing; supervision; formal analysis; investigation; methodology; project administration.

## CONFLICT OF INTEREST STATEMENT

The authors declare that the research was conducted in the absence of any commercial or financial relationships that could be construed as a potential conflict of interest.

### PEER REVIEW

The peer review history for this article is available at https://publons.com/publon/10.1002/brb3.3616.

## Data Availability

Some or all datasets generated during and/or analyzed during the current study are not publicly available but are available from the corresponding author on reasonable request.
